# Is Macroporosity Absolutely Required for Preliminary *in Vitro* Bone Biomaterial Study? A Comparison between Porous Materials and Flat Materials

**DOI:** 10.3390/jfb2040308

**Published:** 2011-11-08

**Authors:** Juliana T.Y. Lee, King L. Chow, Kefeng Wang, Wai Hung Tsang

**Affiliations:** 1 Bioengineering Graduate Program, The Hong Kong University of Science and Technology, Clear Water Bay, Kowloon, Hong Kong, China; E-Mail: bokchow@ust.hk; 2 Division of Life Science, The Hong Kong University of Science and Technology, Clear Water Bay, Kowloon, Hong Kong, China; E-Mail: tsangwh@ust.hk; 3 Department of Mechanical Engineering, The Hong Kong University of Science and Technology, Clear Water Bay, Kowloon, Hong Kong, China; E-Mail: fencal@163.com; 4 National Engineering Research Center for Biomaterials, Sichuan University, 29 Wangjiang Road, Chengdu 610064, China

**Keywords:** porosity, calcium phosphate, osteoinductivity, *in vitro*, alkaline phosphatase

## Abstract

Porous materials are highly preferred for bone tissue engineering due to space for blood vessel ingrowth, but this may introduce extra experimental variations because of the difficulty in precise control of porosity. In order to decide whether it is absolutely necessary to use porous materials in *in vitro* comparative osteogenesis study of materials with different chemistries, we carried out osteoinductivity study using C3H/10T1/2 cells, pluripotent mesenchymal stem cells (MSCs), on seven material types: hydroxyapatite (HA), α-tricalcium phosphate (α-TCP) and β-tricalcium phosphate (β-TCP) in both porous and dense forms and tissue culture plastic. For all materials under test, dense materials give higher alkaline phosphatase gene (*Alp*) expression compared with porous materials. In addition, the cell density effects on the 10T1/2 cells were assessed through alkaline phosphatase protein (ALP) enzymatic assay. The ALP expression was higher for higher initial cell plating density and this explains the greater osteoinductivity of dense materials compared with porous materials for *in vitro* study as porous materials would have higher surface area. On the other hand, the same trend of *Alp* mRNA level (HA > β-TCP > α-TCP) was observed for both porous and dense materials, validating the use of dense flat materials for comparative study of materials with different chemistries for more reliable comparison when well-defined porous materials are not available. The avoidance of porosity variation would probably facilitate more reproducible results. This study does not suggest porosity is not required for experiments related to bone regeneration application, but emphasizes that there is often a tradeoff between higher clinical relevance, and less variation in a less complex set up, which facilitates a statistically significant conclusion. Technically, we also show that the base of normalization for ALP activity may influence the conclusion and there may be ALP activity from serum, necessitating the inclusion of “no cell” control in ALP activity assay with materials. These explain the opposite conclusions drawn by different groups on the effect of porosity.

## 1. Introduction

It has been widely accepted that the porosity or pore size of materials influence the cell behavior and also the osteogenesis process [1]. A number of studies have demonstrated this both *in vivo* (Table A1 in the Appendix) and *in vitro* (Table A2 in the Appendix). Blood vessels are the major transport means in animals and they supply the nutrients for the growth of bone. Porous materials with interconnected pores provide the space for the ingrowth of blood vessels and hence allow better formation of bone in the implants. This is one of the major reasons for the necessity of porosity for bone formation in animal models (*in vivo*). Continuing intensive efforts have been made to prepare and characterize highly porous scaffolds for bone substitute applications [2,3,4,5,6,7,8,9,10,11,12,13] and also assess cellular behavior on these materials [4,7,10,12,14].

Apart from different structures, novel materials of different chemistries are also being developed to have better biocompatibility, osteoconductivity, osteoinductivity, biodegrdation, *etc.* Ideally, it seems it is the best to compare highly porous materials of different chemistries with exactly the same structural properties such as porosity and pore size. Nevertheless, the precise control of porosity of porous ceramics with high interconnectivity was a challenge [15]. A number of recent studies reported on fabrication technologies to synthesize highly porous scaffold of relatively well defined structures [16,17,18,19,20,21] but most require a certain extent of optimization for each material type. For example, there is significant shrinking of the printed scaffolds during the sintering process. Thus the change in dimensions needs to be precalculated by computer assisted design (CAD) [17]. Besides, these methods require more expertise and equipment compared with dense flat materials which can be prepared relatively simply by pressing the powder together by hydraulic press. Thus it is essential to know whether porosity is absolutely necessary in *in vitro* studies, especially for comparative preliminary study of materials with new chemistries.

To address this question, we compared the osteoinductivity of dense materials with porous materials using C3H/10T1/2 cells. Osteoinductivity, which is the ability of a material to induce undifferentiated cells into the osteo-lineage [15,22], is an important property for bone substitute materials. Also totipotent, pluripotent or multipotent cells are required to study this property. Adult stem cells, such as mesenchymal stem cell (MSC) model is a good candidate due to its multipotency, relative ease of maintenance and lack of ethical concerns [23,24,25,26]. We used C3H/10T1/2 cells as they possess the properties of MSCs yet it is an established cell line that may provide more reproducible results as compared to commonly used primary stem cells as its purity is difficult to be controlled precisely in different batches of experiments.

On the other hand, Kaplan *et al.* suggested that higher porosity and pore size result in greater bone ingrowth *in vivo* and lower porosity stimulates osteogenesis by suppressing cell proliferation and forcing cell aggregation *in vitro* [1]. In order to evaluate the effect of cell density on the differentiation of cells *in vitro*, we carried out Bradford protein assay and alkaline phosphatase enzymatic assay for C3H/10T1/2 cells plated with different cell densities.

In short, we compared the *Alp* gene expression of 10T1/2 cells cultured on porous and dense ceramics (hydroxyapatite, HA, α-TCP and β-TCP) and tissue culture plastic. Furthermore, we assessed the effect of cell plating density on ALP expression. The reasons for the different conclusions drawn by different groups were also investigated.

## 2. Experimental Section

### 2.1. Material Synthesis

HA, α-TCP and β-TCP were synthesized by foaming method using 5% hydrogen peroxide (H_2_O_2_) solution [27]. The dried calcium phosphate cylinders were sintered using a programmed oven and then cut using a diamond saw using water as the coolant. The discs have a diameter of 10 mm and a thickness of 1 mm. The average porosity estimated from the Archimedes drainage method was about 71–77%. The dense flat ceramics were prepared by pressing powder together in a mold with 15 mm inner diameter using a hydraulic press and sintered according to the temperature profile in the Table A3 in the Appendix. The sintered discs were trimmed into 10 mm discs by cutting the periphery to obtain discs of the same size.

### 2.2. Material Characterization and Sterilization

The phases of different material surfaces were identified using an X-ray diffractometer (PW1830, Philips). Measurements were performed using a Cu-Kα X-ray source and the XRD spectrum matched with the database using X'Pert HighScore Plus version 2.0. SEM images were captured using scanning electron microscope (SEM) (JSM-6390, JEOL, Japan). The surface porosities were estimated from the SEM photos using the software OriginPro (details were shown in Section 1 of the Appendix) and the surface crater sizes were measured in Photoshop. The discs of materials were sterilized by autoclaving at 121 °C for 20 min and dried in an 80 °C oven.

### 2.3. Cell Subculturing

C3H/10T1/2 cells (ATCC, CCL-226™) were cultured in 10cm culture dishes (Nunc) containing Dulbecco's Modified Eagle Medium, DMEM (Invitrogen) supplemented with 10% fetal bovine serum (FBS) and antibiotics (100 U/mL penicillin and 100 μg/mL streptomycin) in an incubator at 37 °C under a humidified atmosphere of 95% air and 5% CO_2_. At 95% confluence, the cells were trypsinized, resuspended in complete medium and counted in a hemocytometer. Then the cells were plated for the assays and medium changed at every three days.

### 2.4. RT-qPCR with Cells from Porous Materials and Flat Materials

9 × 10^4^ cells were seeded on each disc in 48-well plates (Nunc). Wells without materials added (plastic surfaces) were used as controls. RNA from the cells on the material was extracted by TRIzol at day 6 according to the manufacturer's instruction. For cells on α-TCP and β-TCP, modifications were made to the RNA extraction protocol as described previously [28]. Crushing the porous materials in TRIzol was performed for the effective extraction of RNA from cells in the pores of porous material while this is not necessary for dense materials since the cells cannot grow into the dense materials. The subsequent RNA quality check, yield estimation, DNase1 treatment, reverse transcription and qPCR on *Alp* and *β-Actin* (the house-keeping gene) were also performed as described [28]. (In this report, “ *Alp* ” here denotes the gene that codes for alkaline phosphatase in mouse and “ALP” denotes the alkaline phosphatase protein according to the gene nomenclature convention adopted in the biology field. On the other hand, the alkaline phosphatase gene in human is generally denoted by “ALP”).

### 2.5. ALP Enzymatic Assay with Different Cell Plating Densities

Different numbers of cells (1 × 10^4^, 2 × 10^4^ and 4 × 10^4^) were plated in a 96-well cell culture plate (Nunc) at different time so that the assays would be performed with cells after 2 and 7 days of culturing for ALP enzymatic assay and 2, 5, and 7 days of culturing for Bradford protein assay. Cells were washed twice with PBS and lysed with 0.2% Triton X-100 solution. The lysate was mixed with 1 mg/mL *p*-nitrophenyl phosphate (*p*-NPP, Sigma) in 0.1 M glycine buffer (pH 10.4) containing 1 mM MgCl_2_, 1 mM ZnCl_2_. Absorbance at 405nm was measured after incubation in dark and the amount of *p*-nitrophenol (*p*-NP, Sigma) formed was calculated based on a standard calibration curve of *p-*NP. Bradford assay was also performed using the same lysate and the absorbance value was read at 595 nm. Protein amount was calculated based on a standard calibration curve of bovine serum albumin (BSA). Phase contrast photos of cells were captured the day before the assays.

### 2.6. SEM of Cells on Scaffolds

Cells on porous HA were prepared for scanning electron microscope (SEM) examination using procedures described previously [29]. In brief, C3H/10T1/2 cells were plated on porous HA at 9 × 10^4^ cells per cm^2^. After 1 week, the porous scaffolds with cells were blotted dry with absorbent papers and washed with phosphate buffered saline (PBS), pH 7.4. They were then fixed with 2.5% glutaraldehyde in PBS, pH 7.4 (for resorbable calcium phosphate, 0.05 M cacodylate buffer, pH 7.4, should be used instead). The scaffolds were rinsed twice with PBS and then with double deionised water (DDI) with subsequent quick freeze in liquid nitrogen and transferred to a freezer dryer for overnight drying. Scanning electron microscope (SEM) (JSM-6390, JEOL, Japan) was utilized to observe the morphology of cells on the porous HA.

### 2.7. DAPI Staining

9 × 10^4^ cells were seeded on each porous HA disc in 48-well plates (Nunc). At day 3, the discs were washed with PBS and then fixed by 4% paraformaldehyde for 5 min at 4 °C. The permeability of cells on the discs was increased by washing 3 times in 0.1% Triton X-100. 4′,6-diamidino-2-phenylindole (DAPI) stain was applied to the discs at 1 μg/mL in 0.1% Triton X-100 for 5 min, followed by washing with Triton X-100. Fluorescence images were taken using Olympus upright fluorescence microscope BX41. 5 to 15 images were taken for each spot due to the limitation of the depth of field of the microscope and cells lying at different depth. The photos at different depth were merged using ImageJ with the “Extended depth of field” plug-in.

### 2.8. Statistical Analysis

P-values were calculated using Student's two-tailed t-test of unequal variance.

## 3. Results

### 3.1. Material Characterization

**Figure 1:**
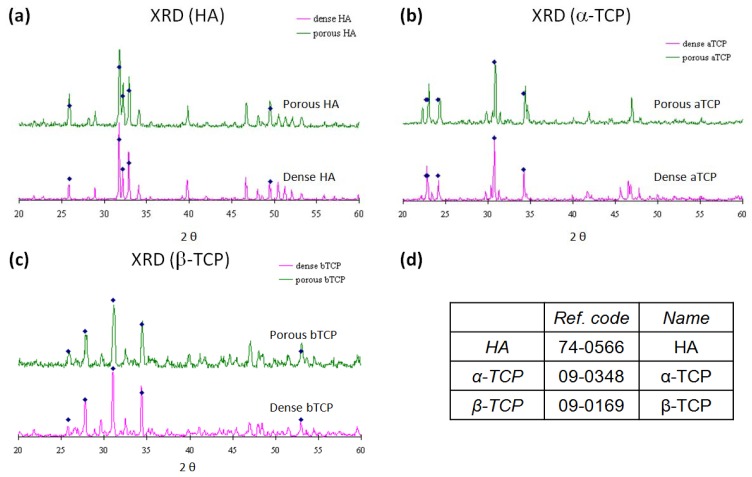
XRD spectra of porous and dense: (**a**) hydroxyapatite, HA; (**b**) α-TCP and (**c**) β-TCP discs; and (**d**) the corresponding matches from the database (the spots on the spectrum indicate the positions of the five most intense peaks from the powder X-ray data of the database).

**Figure 2:**
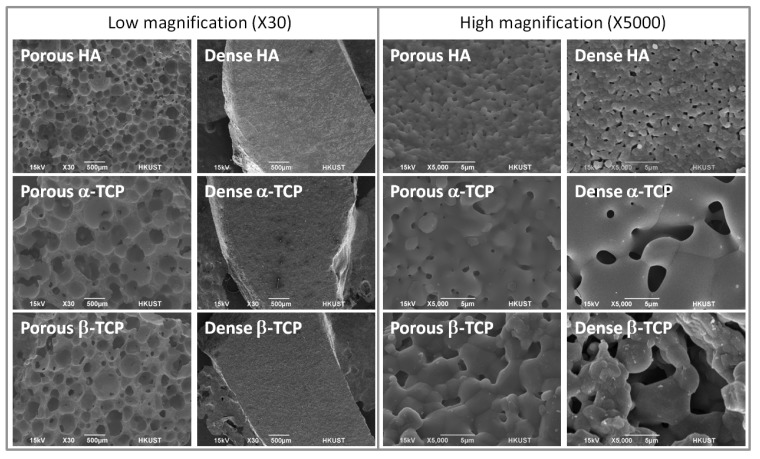
Surface topography of porous materials and dense materials (HA, α-TCP and β-TCP) revealed by SEM at 30× and 5000×.

The identity of the materials was confirmed by XRD and morphology was viewed by SEM. Figure 1 shows the XRD spectra of the chosen ceramics. The peaks of the spectra match the most intense peaks in the database and the identity of materials was confirmed. There is no significant difference between the spectra of materials in dense forms and porous forms. The porous materials used are highly porous with considerable degree of interconnections among the pores while the dense materials have flat surfaces as shown in Figure 2. From Table 1, the surface porosities of the three materials are similar but the surface crater sizes are different with HA having the smallest craters while α-TCP the largest. The porous HA, α-TCP and β-TCP materials in this study were made by the same H_2_O_2_ foaming method. Yet there is a difference in pore morphology, which resulted from different degrees of shrinkage during the sintering process. To obtain precisely controlled pore sizes, it is necessary to carry out optimization experiments to estimate the shrinkage ratio during the sintering process and also trialed with different synthesis parameters.

**Table 1:** Surface porosity and crater size of the porous HA, α-TCP and β-TCP (average ± SD).

	**Porous HA**	**Porous α-TCP**	**Porous β-TCP**
Surface porosity ^a^ (%)	67.3 ± 1.7	68.1 ± 5.8	66.5 ± 1.5
Crater size ^b^ (μm)	203 ± 88	342 ± 134	269 ± 111

a n = 4 photos;

b n = 60 measurements.

### 3.2. Yield of RNA from Cells on Materials

From Table 2, the amount of RNA extracted from cells on the porous discs is significantly higher than that from dense discs of the same cross sectional area (p < 0.005). This indicates a larger number of cells are present on the porous materials compared with the dense materials. As the same number of cells was plated initially and assuming the dense materials do not induce much higher death rate, there was a larger proportion of cells attached on the porous materials initially and/ or higher proliferation of cells on the porous materials compared with dense materials.

**Table 2:** Yield of RNA (μg) extracted from cells on porous calcium phosphate (CaP) materials, dense flat CaP materials and flat tissue culture plastic.

	**Porous CaP** a	**Flat CaP** a	**Flat plastic** b
Average	3.47	1.80	2.63
SD	0.89	0.17	0.39

a n = 6 with 2 discs from HA,α-TCP and β-TCP each;

b n = 2.

### 3.3. Alp Gene Expression

**Figure 3:**
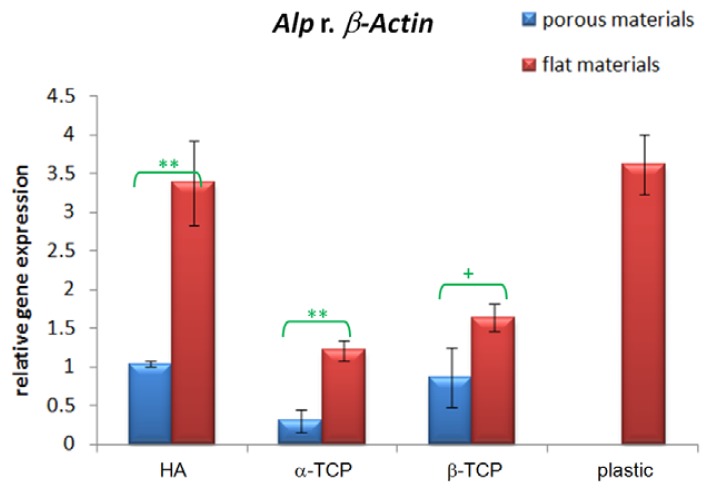
Relative quantification of gene expression of *Alp* normalized with *β-Actin* of 10T1/2 cells seeded on HA, α-TCP, β-TCP and plastic after 6 days of culture (n = 5, technical duplicate in batch 1 and triplicate in batch 2; mean ± SEM; ** p < 0.01, ^+^ p < 0.1).

As shown in Figure 3, the *Alp* mRNA level of 10T1/2 cells was highest when cultured on HA scaffolds. For dense flat materials, the *Alp* mRNA level was slightly higher for cells on β-TCP than α-TCP while for porous materials, the cells on β-TCP has much higher *Alp* expression than α-TCP but not statistically significant. Cells on all calcium phosphate materials under test have lower *Alp* expression compared with cells on flat tissue culture plastics. There were consistent higher *Alp* mRNA levels for cells on dense flat materials compared with porous materials (p < 0.01 for HA and α-TCP and p < 0.1 for β-TCP).

### 3.4. Effect of Cell Density

**Figure 4:**
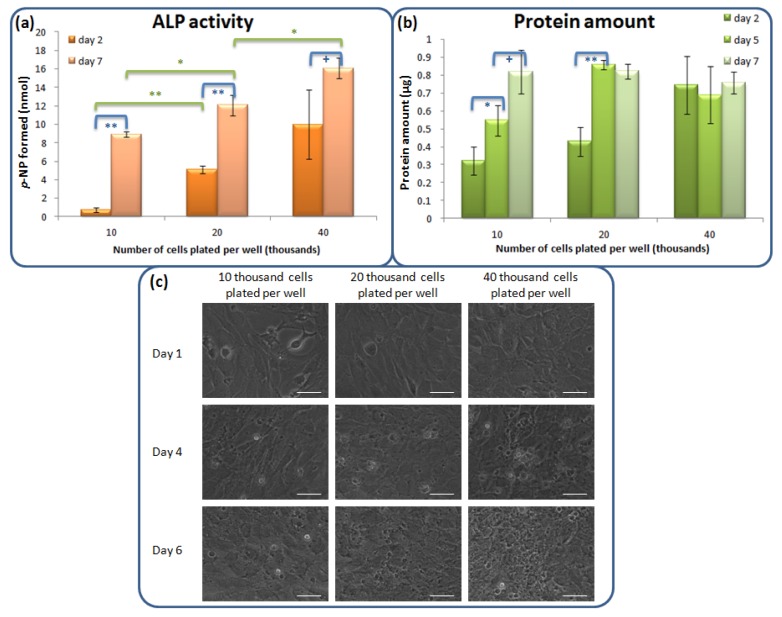
Effect of cell plating density on ALP expression and total protein amount of 10T1/2 cells seeded on a plastic culture plate (**a**) ALP enzymatic activity estimated from *p*-NPP assay after 2 and 7 days of culture, with background subtraction using values obtained in the “no cell” control (n = 3 wells; mean ± SD; ^+^ p < 0.1, * p < 0.05, ** p < 0.005); (**b**) Protein amount after 2, 5 and 7 days of culture estimated from Bradford assay (n = 3 wells; mean ± SD; ^+^ p < 0.1, * p < 0.05, ** p < 0.005); (**c**) Phase contrast photos of 10T1/2 cells seeded on a 96-well plastic culture plate with different plating densities and photos captured the day before the assays (*i.e.*, at day 1, 4 and 6) (scale bar =50 μm).

In order to verify whether the alkaline phosphatase expression of 10T1/2 cells is affected by cell aggregation, experiments with cells plated on plastic at different densities were performed. From Figure 4a, the ALP activity of cells increases from day 2 to day 7, indicating the cells become more differentiated. The ALP activity is higher for cells plated at higher initial plating density after both 2 days and 7 days of culturing. Higher initial plating densities also gave higher *Alp* mRNA levels (data not shown). From Figure 4b, the protein amount in cells increases with time for low plating density but does not significantly change for high plating density. At day 2, the protein amount is larger for cells plated at higher density. Figure 4c shows the photos of cells captured a day before the assay. The cells increase in number for all initial plating densities from day 1 to day 6. Our results are consistent with the phenomenon that proliferation precedes differentiation in general [30,31]. When the cells are plated at higher cell density, they have less room for proliferation (as shown in Figure 4c and deduced from Figure 4b) and would get into the differentiation program earlier (as shown by the higher ALP activity in Figure 4a).

### 3.5. Distribution and Morphology of Cells in Porous Scaffolds

**Figure 5:**
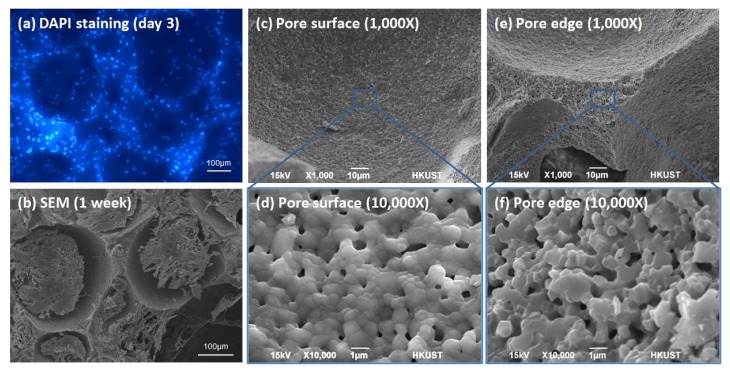
(**a**) 10T1/2 cells viewed under a fluorescent microscope with DAPI staining the nuclei of cells cultured for 3 days; (**b)** SEM photos of 10T1/2 cells cultured on porous HA for 1 week; SEM photos of (**c**) pore surface at 1,000×; (**d**) pore surface at 10,000×; (**e**) pore edges at 1,000× and (**f**) pore edges at 10,000×.

The cell density on the edges of the pores observed by DAPI staining is higher than the pore surface (Figure 5a) and this may be due to the rougher surface of the edges (Figure 5e and f compared with Figure 5c and d) caused during slicing of blocks into discs. From the SEM photos, the cells in the porous HA span across the pores after one week of culture and only the “outermost” layers of cells may be in direct contact with the material (Figure 5b). The gene expression may be different for cells directly attaching on the material compared to the spanning cells and the percentage of the cells that are directly touching the materials is probably different for different pore sizes and porosities. For most biochemical assays, they are monitoring the overall level of a cellular component of the cell mass instead of an individual cell. Therefore, difference in the actual distribution and arrangement of cell mass may be one of the reasons for the observed difference in *Alp* mRNA levels of cells seeded on scaffolds with different geometries but with the same chemistry.

## 4. Discussion

### 4.1. Effect of Macroporosity of Calcium Phosphate Discs

A number of studies have investigated the effect of porosity on bone growth *in vivo* and cell proliferation *in vitro*, but few studies have focused on the osteoinductivity *in vitro*. Thus this study focused on osteoinductivity of three commonly used calcium phosphate materials: HA, α-TCP and β-TCP. In order to have the largest difference, we compared the extremes: dense materials without macroporosity and highly porous materials prepared by foaming. Among several possible osteomarkers, we chose to assess the *Alp* mRNA level as *Alp* expression increased with time but was consistent at different time points in our separate experiments [32]. The results in this study show that the cells on dense flat materials have higher *Alp* expression compared with porous materials for HA, α-TCP and β-TCP (Figure 3). This is consistent with the conclusion drawn by Kaplan *et al.* from other materials that lower porosity stimulates osteogenesis by suppressing cell proliferation and forcing cell aggregation in *in vitro* systems [1]. Tabata *et al.* demonstrated with non-woven fabrics that osteogenesis *in vitro* is enhanced by lower porosity [33]. Although porous materials are highly preferred in bone substitute application and cellular behavior on these materials were intensively studied [4,7,10,12,14], our results suggest that high porosity may not be absolutely required for *in vitro* osteogenesis assay. As variations in porosity of materials may affect the response of cells on them, dense flat materials may be used for preliminary *in vitro* osteoinductivity study, especially when it is difficult to have precise control of material porosity for a particular synthesis method. In Figure 3, the consistent trend of *Alp* mRNA level (HA > β-TCP > α-TCP) between porous materials and dense materials also justifies the use of dense materials in preliminary study even though highly porous materials have been used in many studies due to *in vivo* consideration.

On the other hand, the difference of cell response in 2D and 3D settings has been extensively studied using different models with examples tabulated in Table A4 in the Appendix. It is well accepted that cells in monolayer behave differently compared with 3D cultures or on 3D scaffolds. As shown in Table A4 of the Appendix, 3D culturing can influence the cell attachment, viability, proliferation, gene expression, differentiation, cellular response to chemicals, biomolecule synthesize and maturation. Nevertheless, in most studies, the cells were cultured on substrates with different chemistries in the 2D and 3D settings or the materials on which the cells were cultured were not mentioned explicitly. In this report, we compared the *Alp* expression of cells on planar surfaces and 3D scaffolds of the same substrate material chemistry.

### 4.2. ALP Activity Assay

Though our result agrees with the postulation that lower porosity enhances osteogesis observed for other materials [1], it is opposite to the conclusion of a study using HA with different porosities [34]. In our study, we measured the *Alp* mRNA level normalized with *β-Actin* mRNA level while the previous study assessed the ALP activity per disc.

**Figure 6:**
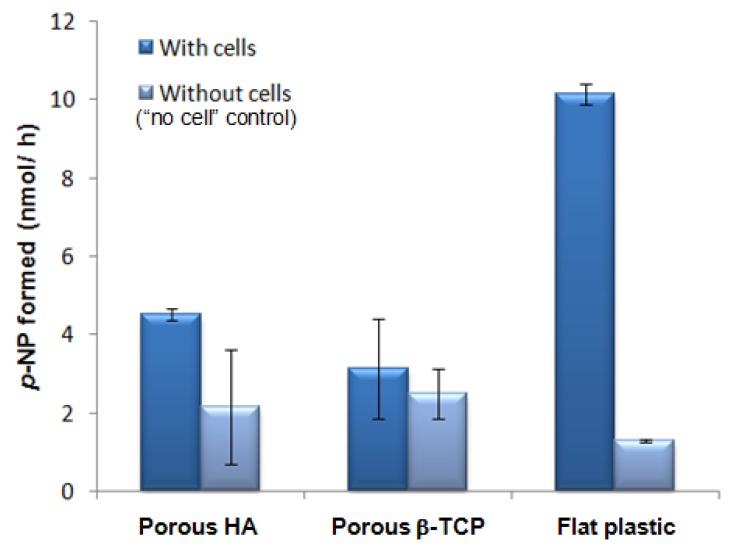
ALP activity of cells on materials detected using ALP enzymatic assay (9 × 10^4^ cells plated per well of a 48-well plate, assayed after 3 days of culture; n = 2; The experiment was repeated and the high background ALP activity detected for the “no cell” control of porous materials was consistently observed in the repeated experiments).

We did not draw a conclusion from ALP activity assay in this report since there was high ALP activity for calcium phosphate samples even for “no cell” control (all conditions were the same except that cells were not added and supposed not to give ALP activity) (Figure 6). Besides this, we investigated whether cell culture medium may contribute to the detected ALP activity with results further discussed in Section 2 of the Appendix. ALP enzymatic assay is widely used in the biomaterial field and this study does not suggest the enzymatic assay cannot be used for biomaterial study. In contrast, we would like to emphasize whether this assay is applicable for a certain type of material depends on the relative background ALP activity because of ALP adsorption from serum, which can be estimated through the use of “no cell” control. This background signal is influenced by various factors such as the material affinity for ALP, the material geometry (e.g., higher background when cell culture medium with serum is easily trapped inside the inner pores and not easily removed during the washing step) and also the pre-treatment before cell plating (e.g., fibronection coating which can enhance cell adhesion and may block ALP adsorption at the same time which would reduce the background). The results are more reliable when the ALP activity in the “no cell” control is much lower than the samples under study.

On the other hand, different numbers of cells can be present on materials of different porosities in the same volume of disc. Thus we hypothesized that the difference in the base of normalization may be one of the reasons for the contrary conclusions. As shown in Table 3, the ALP activity increases with increasing plating density when normalized by protein amount or area covered by the cells but decreases with increasing plating density when normalized by the initial cell number. This shows that the choice of normalization base would affect the conclusions drawn and this is further discussed in Section 3 of the Appendix.

**Table 3:** Comparison of ALP activity of 10T1/2 cells cultured in a 96-well plate for 7 days and normalized by different attributes.

**Number of cells plated per well of a 96-well plate**	**10,000**	**20,000**	**40,000**
Cell plating density (thousand cells/sq cm)	31.3	62.5	125
*p*-NP formed (nmol)	8.92 (±0.26)	12.06 (±1.10)	16.08 (±1.10)
Normalized by initial cell number (nmol/thousand cells)	0.89 (±0.03)	0.60 (±0.05)	0.40 (±0.03)
Normailzed by protein (nmol/mg protein)	10.91 (±1.66)	14.68 (±1.53)	21.25 (±2.26)
Normalized by area covered (nmol/sq cm)	27.87 (±0.81)	37.68 (±3.43)	50.25 (±3.43)

### 4.3. Tradeoff between Higher Clinical Relevance and Lower Variation

For the final clinical application, it seems the best to simulate the *in vivo* environment in human as closely as possible even for cell culture system. This would imply porous scaffolds should be used in experiments for bone substitute application since porous materials allow blood vessels to grow into the materials which are essential for bone formation in large bone substitutes. Numerous studies have demonstrated successful bone formation in their porous materials to show the novel materials are suitable for bone substitute application. However, in order to show the new material is better than the widely used materials, it is essential to compare with a common reference material. (The use of a reference material or control was discussed in our previous report [32]). Proper scientific control is also required. In other words, it would involve the comparison of cell responses to at least two different materials. To study new material's chemistry or microstructure, the material geometry and structure at macro-scale should be as close as possible for all the materials under study.

**Figure 7:**
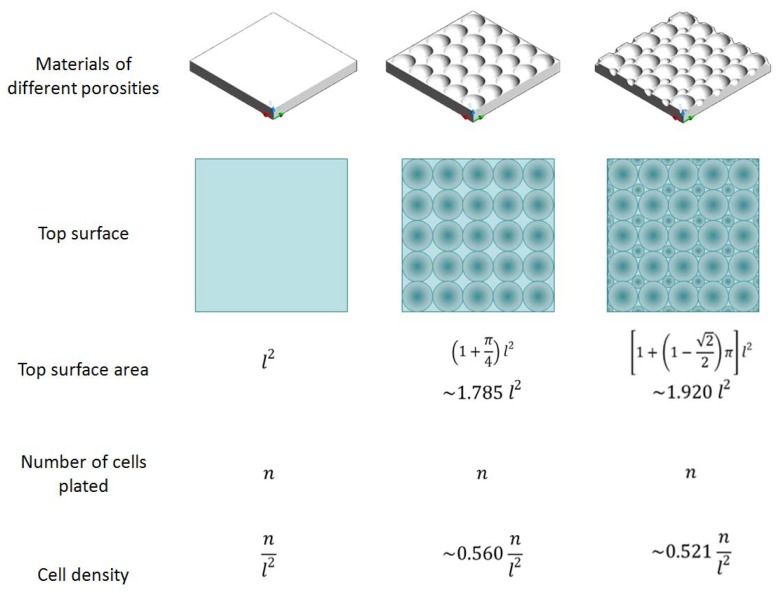
Schematic showing the relation between porosity, surface area and cell density.

Materials with different porosities may have differences in surface area and cell densities (Figure 7). Considering the top surface alone, the surface area of a highly porous material can be 0.92 times larger than flat material, causing considerable difference in cell density. The difference would be even greater if inner pores were also taken into account. From Figure 4, difference in initial cell density can result in significant difference in osteo-marker expression of 10T1/2 mesenchymal stem cells. Therefore, comparable porosity is required for reliable conclusion. Besides, this variation in porosity in replicate samples would cause variation in the gene expression of cells cultured on them, thus may hinder statistically significant conclusions to be drawn. This study validates the use of dense flat materials for preliminary study when precisely defined porous structures are not easily available.

On the other hand, the number of cells to be seeded on the scaffolds may be adjusted in consideration of the actual available surface on the material for cell attachment and growth. For instance, more cells might be seeded on materials with larger surface area to minimize the variations introduced because of the cell density difference. Yet as mentioned in the result section, the actual distribution and arrangement of cell mass may affect the observed difference in *Alp* mRNA levels. Besides, it may also be technically difficult to adjust the number of cells to be seeded accurately since the surface of different discs may vary even if they are synthesized in the same batch. Hence, it may be possible to reduce the effect of cell density difference by adjusting the initial cell seeding density based on the surface area of materials, but this is not likely to eliminate the problem completely.

**Figure 8:**
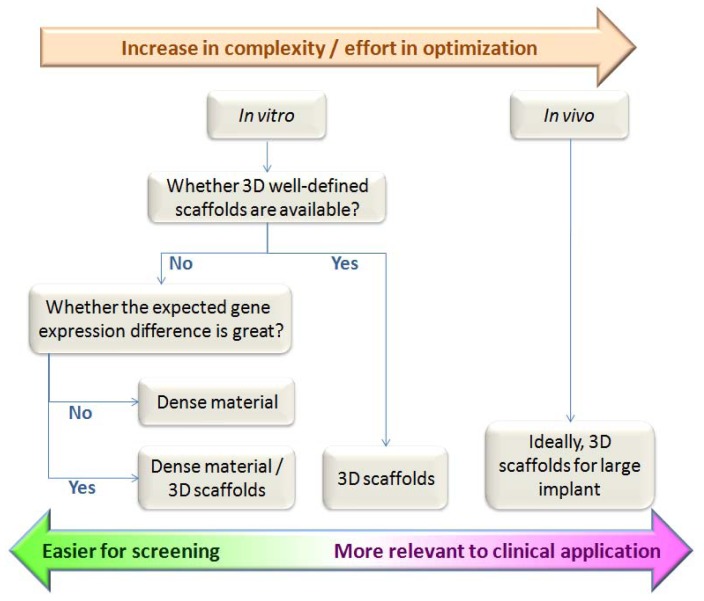
Factors involved in deciding scaffold type.

Minimizing the variations and improving the reproducibility of experiments are very important in preliminary *in vitro* study for accurate and reliable conclusions. This lays the foundation regarding the choice of materials for further *in vivo* experiments which are costly and with limited number of samples that can be handled due to cost and ethical issues. In this study, we have investigated three different materials of single phase (HA, α-TCP, β-TCP). In the actual application, biphasic or even triphasic materials are under intensive research. Besides, new materials with a slight difference in chemical compositions such as metal substitution of calcium in the hydroxyapatite are also developed to optimize the bone formation. Thus a continuum of materials instead of single phase materials may need to be compared and the difference among materials may not be as great as those among materials of single phase. This necessitates the stringent minimization of variations due to geometry, porosity, *etc.* Hence, although porous materials seem to be better in term of clinical relevance, it may not be always desirable to use porous materials in preliminary *in vitro* assays when the scaffolds geometry cannot be controlled precisely among the materials under study (Figure 8).

## 5. Conclusions

In contrast to many studies comparing cell behavior of cells on 3D materials with cell monolayer on plastic surface, this study compares cells on flat materials with 3D porous materials of the same chemistry. More cells are present on the porous materials compared with dense flat materials when the same number of cells was initially plated. Flat materials give higher osteo-marker expression of cells than porous materials as proliferation is suppressed and differentiation enhanced, which is supported by the higher ALP expression of cells with higher initial cell plating density. On the other hand, porous materials have a larger surface area which would result in lower initial cell density compared with dense flat materials. Hence, for reliable comparison, it is necessary to use materials of comparable porosities in evaluating materials of new chemistries.

Despite the significant difference in the *Alp* expression between porous and flat materials, a consistent trend of *Alp* mRNA level (HA > β-TCP > α-TCP) was observed for both porous and flat materials. This suggests that flat materials could be used for preliminary comparative study of materials with different chemistries to avoid variations in the gene expression due to porosity difference. There is often a tradeoff between higher clinical relevance and less experimental variations in the experiments. Dense flat materials may allow quicker and more reliable early assessment of materials with new chemistries.

This study also suggests that the base of normalization for the ALP enzymatic assay may influence the conclusions drawn. Besides, “no cell” controls are required for reliable conclusions due to the possible ALP adsorption on the materials from the serum. These two technical aspects suggest possible explanations to the contrasting findings from different groups.

## Figures and Tables

**Figure A1:**
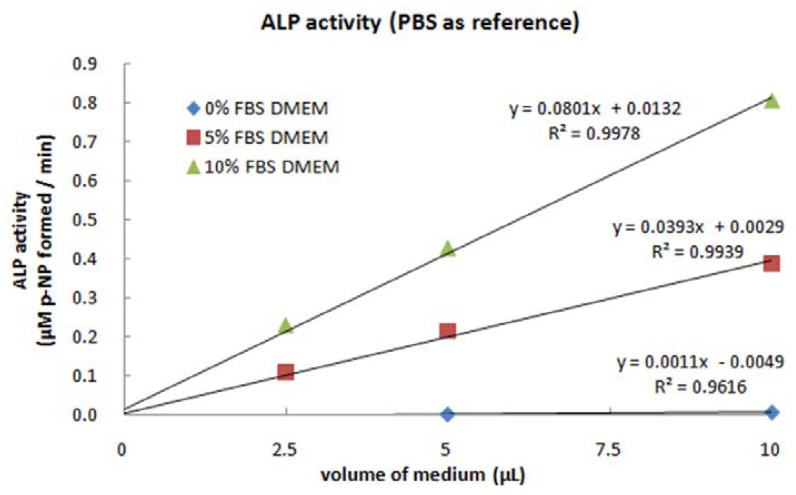
ALP activity of different dilution of cell culture medium (2.5 μL, 5 μL and 10 μL) of different serum concentration (0%, 5% and 10% FBS) with PBS as the reference.

**Figure A2:**
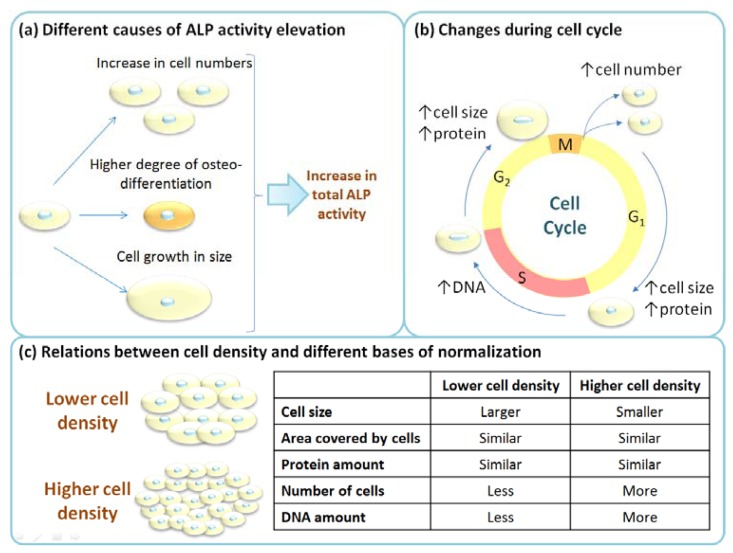
(**a**) Different causes of ALP activity elevation; (**b**) Events of a mammalian cell cycle; (**c**) Relations between cell density and the bases of normalization (area coverage, protein amount, cell number and DNA amount).

## Appendix

**Table A1:** *In vivo* cell response for cells seeded on 3D scaffolds with different porosities or pore sizes from literatures.

**Material** a	**Implant site**	**Porosity (%)**	**Macropore size (μm)**	**Specific surface area (m^2^/g)**	**Assessments** b	**Conclusion** a,b	**Reference**
BCP	In femurs of rabbits	40, 50%	300, 565	/	Histology	Macroporous biphasic calcium phopshate implants with 565 μm pore diameter and 40% macroporosity represented the optimal association for homogeneous and abundant bone ingrowth.	[62]
BCP	Intramuscularly in goats	macroporosity: 58%; microporosity: 4–24%	<∼400	0.2–9.7	Histology and histomorphometry	The increased specific surface area led to more surface reactivity, which is hypothesized to be essential for osteoinductivity by biomaterials.	[63]
BCP	Intramuscularly in goats	70, 75%	/	0.2, 1.5	Histology and histomorphometry	The BCP scaffold with 75% porosity performed better than the one with 70% porosity in both the amount and rate of bone formation.	[64]
β-TCP	In drill hole defects in cancellous bone of sheep	/	150, 260, 510, 1220	/	Macroscopy, histology and histomorphometry	Samples with an intermediate macropore size (510 *μ*m) were resorbed significantly faster than samples with smaller macropore sizes (150 and 260 *μ*m). However, this fast resorption was associated with a lower bone content and a higher soft tissue content.	[65]
β-TCP	Subcutaneously in mice	25, 65, 75%	/	0.12, 0.18, 0.3	Specific ALP activity, histology	A higher porosity of TCP scaffolds does not necessarily mean a higher ALP activity *in vivo*. The distribution and size of the pores, as well as the surface structure, might play an important role for osteogenic differentiation *in vivo*.	[66]
HA	Intramuscularly in goats	60, 70%	400, 800	/	Histology and histomorphometry	The microporous HA scaffolds with 70% porosity and 800 *μ*m pore size yielded more bone than did HA scaffolds with 60% porosity and 400 *μ*m.	[67]
HA	Subcutaneously in mice	65, 80%	/	0.87, 1.63	Histology	The porosity and pore interconnection of osteoconductive scaffolds can influence the overall amount of bone deposition, the pattern of blood vessels invasion and finally the kinetics of the bone neoformation process.	[68]
HA	Subcutaneously in rats	30, 50, 70%	/	/	Histology, ALP acitivity and OCN content	Expanded bone formation was observed earlier for constructs with higher porosity. The ALP activity and OCN production also increased with increasing porosity.	[34]
HA	Subcutaneously in rats	70%	106–600	0.024	Histology, ALP acitivity and OCN content	The ALP activities at 2 wk and the OCN contents at 4 wk after implantation was the highest in the ceramics implants with pore size of 300-400 *μ*m.	[69]
HA	Subcutaneously in rats	Tunnels of 0.7mm or 3mm in diameter	/	/	Histology, Ca and Type II collagen content	The “vasculature-inducing geometry” of the carrier as an extracellular matrix is crucially important for osteogenesis.	[70]
HA, BCP	Intramuscularly in goats	46.5–54.3%	243.9–380.2	0.07–1.60	Histology and histomorphometry	Histomorphometrical results showed that the presence of micropores within macropore walls is necessary to make a material osteoinductive.	[71]
HA, TCP	In cancellous bone of rabbits	60%	50–100 and 200–400	/	Histology	For TCP, bone and tissue ingrowth and implant resorption occurred at a higher rate in the smaller-pored materials compared with the larger-pored ones. For HA, the smaller-pored materials was totally infiltrated by bone or bone marrow after four months but not for the larger-pored ones.	[72]
Ni-Ti alloy	In femurs of rats	46.6, 59.2, 66.1	259, 272, 505	/	Histology and histomorphometry	The porosity of 66.1% showed best bone contact (51%) of the porosities tested.	[73]
PCL	Subcutaneously in mice	/	350, 550, 800	/	Histology, μCT and mechanical testing	Pore sizes between 350 and 800 μm play a limited role in bone regeneration in this tissue engineering model.	[74]
PPF	In cranial defects in rabbits	57–75%	300–500, 600–800	/	Light microscopy, histological scoring analysis and histomorphometric analysis	Scaffold porosity and scaffold pore size were not found to significantly affect the observed tissue response.	[75]
Ti_6_Al_4_V	In femurs of rabbits	/	100, 200, 300	/	Histology and histomorphometry	200 μm may be the optimal pore size for laser-textured Ti_6_Al_4_V implants.	[76]
Ti_6_Al_4_V	In femurs of rats	3, 11, 25%	/	/	Ca concentration	25% porosity samples showed the highest amount of calcium concentration within the pores, suggesting a faster rate of tissue generation and integration compared to samples with lower pore volume.	[77]

a BCP: biphasic calcium phosphate, β-TCP: β-tricalcium phosphate, HA: hydroxyapatite, PCL: polycaprolactone, PFF: poly-(propylene fumarate);

b ALP: alkaline phosphatase, OCN: osteocalcin, μCT: micro-computed tomography.

**Table A2:** *In vitro* cell response for cells seeded on 3D scaffolds with different porosities or pore sizes from literatures.

**Material** a	**Cell**	**Porosity (%)**	**Macropore size (μm)**	**Specific surface area (m^2^/g)**	**Assessments** b	**Conclusion** a,b	**Reference**
β-TCP	Human mesenchymal stem cells	25, 65, 75%	/	0.12, 0.18, 0.3	Protein production, specific ALP activity	*In vitro* porosity was beneficial for protein production, but did not influence osteogenic differentiation.	[66]
CO_3_Ap-collagen sponges	MC3T3-E1 cells	48.9, 72.6, 79.2%	50–300	/	Histology	There was no significant difference in the invading osteoblast quantity for composite of apatite and collagen with pores ranging from 50–300 μm and porosities of 49–79%.	[78]
COL-CG	MC3T3-E1 cells	/	85–325	0.00221–0.00845 μm^−1^	Cell adhesion and infiltration	Scaffolds with a mean pore size of 325 μm were deemed optimal for bone tissue engineering.	[79]
HA	Rat primary bone marrow mesenchymal stem cells	30, 50, 70%	/	/	ALP acitivity and OCN content	The ALP activity and OCN production increased with increasing porosity.	[34]
HA	Primary human osteoblast-like cells	67-76%	/	/	SEM	The introduction of microporosity has no evident effect on cellular morphology at later time points but it seems to play a role in initial cellular anchorage and attachment.	[80]
HA	Rat bone marrow cells	<5, 15, 30%	/	/	Cell attachment, proliferation, total protein content, ALP activity and bone-like nodule formation	The intermediary and final events such as proliferation, protein synthesis, ALP activity, and bone-like nodule formation favored surfaces with a more regular topography, such as that presents in HA with 15% or less of microporosity.	[81]
HA	MC3T3-E1 cells, L132 cells	0.2, 18.3, 25, 80%	1–10, 10–50, 500–600	/	Cell proliferation, viability, SEM, cytochemical staining	The micro-porous HA (internal pore size of 1–10 μm) induced the highest cell growth.	[82]
HA	Autologous human mesenchymal stem cells	75, 88%	200, 500	/	DNA content, ALP activity, histology, SEM and RT-qPCR	The 200-μm pore scaffolds exhibited a faster rate of osteogenic differentiation than did the 500-μm pore scaffolds.	[53]
PCL	Rat marrow stromal cells	84-89%	20–45	/	Cell attachment, spreading and histology	Increasing the thickness of the nanofiber layer (also increasing the pore size) reduced the infiltration of cells into the scaffolds.	[83]
PET	Mesenchymal stem cells	92.9-97.0%	/	/	Cell proliferation, ALP activity, OCN content and SEM	The attachment, proliferation and bone differentiation of MSC was influenced by the fiber diameter and porosity of non-woven fabrics as the scaffold.	[33]
TiO_2_	Human bone-derived cells	/	0.4, 13, 49	/	Cell proliferation	Smaller pores (0.4 and 13 μm) in TiO_2_ films enhanced the proliferation of human cells trypsinised from bone in contrast to larger pores (49 μm).	[84]

a β-TCP: β-tricalcium phosphate, COL-CG: collagen-glycosaminoglycan, HA: hydroxyapatite, PCL: polycaprolactone, PET: polyethylene terephthalate;

b ALP: alkaline phosphatase, OCN: osteocalcin, SEM: scanning electron microscopy, RT-qPCR: reverse transcription quantitative polymerase chain reaction.

**Table A3:** Sintering temperature profile for the calcium phosphate ceramics.

**HA**		**α-TCP**		**β-TCP**	

Temp. (°C)	Duration (min)	Temp. (°C)	Duration (min)	Temp. (°C)	Duration (min)
		
0–400	160	0–400	160	0–400	160
400	120	400	120	400	120
400–800	160	400–800	160	400–800	160
800	240	800	240	800	240
800–1100	120	800–1250	180	800–1,100	120
1100	120	1250	360	1,100–900	240
normal cooling		quick cooling		900	360
				normal cooling	

**Table A4:** Cell response in 3D culture compared with 2D from literatures.

**Related cell type**	**Cell**	**2D material**	**3D material** a	**Comparison between 2D and 3D** a	**Reference**
Cancer cell	C4-2B cells	Collagen-coated tissue culture plastic	Electrospun collagen membrane	The cells on electrospun substrates were more resistant to both antineoplastic agents, docetaxel, and camptothecin compared to the cells grown on standard collagen-coated tissue culture polystyrene.	[85]
Chondrocyte	Rat bone marrow cells	Monolayer	3D chitosan or chitosan/gelatin scaffolds	The chitosan scaffolds caused a reduction in alkaline phosphatase production and an increase in the collagen concentration indicating phenotypic changes in the cells after the addition of a chondrogenic medium.	[86]
Chondrocyte	Human articular chondrocytes (HAC)	Monolayer on culture plates	Type II collagen sponges	Three-dimensional expansion of HAC on the scaffolds, as compared with 2D expansion for the same number of doublings, better maintained the chondrocytic phenotype of the expanded cells mRNA) but did not enhance their accumulation of glycosaminoglycan (GAG) following chondrogenic culture. Besides, increasing the HAC seeding density in the scaffolds (from 25 × 10^3^ to 66 × 10^3^ cells/mm^3^) significantly improved chondrogenesis (up to 3.3-fold higher GAG accumulation and up to 9.3-fold higher type II collagen mRNA).	[87]
Chondrocyte	Autologous chondrocytes and mesenchymal stem cells (MSCs)	Monolayer	3D pellet or fibrin-sealant construct	There was a proliferative effect for MSCs exposed to PRP in monolayer culture and an increase in the expression of chondrogenic markers when cells are exposed to a 3D environment.	[88]
Chondrocyte	Bovine chondrocytes	Monolayer in flasks	3D Minusheet	The Minusheet cultures usually showed a markedly higher mRNA expression than monolayer cultures. Besides, the ratio of type-I to type-II collagen or aggrecan to type-I collagen remained higher in Minusheet 3D cultures than in monolayer cultures.	[89]
Endothelial cell	Primary human artery-derived fibroblasts and human umbilical vein endothelial cells	Monolayer cell sheets	Self-assembled cell-based microtissues	The microtissue has significant enhancement of ECM expression and maturation.	[90]
Fibroblast	Fibroblast cell line GD25b1	Monolayer	3D matrices derived mouse embryo sections or naturally deposited ECM	The relative content of unsaturated fatty acids, which serve as targets of oxidative attack, was observed to be higher in major phospholipids in plasma membranes of 3D cells.	[91]
Fibroblast	Human foreskin fibroblasts	Glass cover slips coated with different proteins or 3D matrix compressed to form localised 2D matrix	3D matrices derived either from detergent-extracted mouse embryo sections or naturally deposited 3D ECM	3D-matrix adhesions differ from focal and fibrillar adhesions characterized on 2D substrates in their content of α_5_β_1_ and α_v_β_3_ integrins, paxillin, other cytoskeletal components, and tyrosine phosphorylation of focal adhesion kinase (FAK). Relative to 2D substrates, 3D-matrix interactions also display enhanced cell biological activities and narrowed integrin usage.	[92]
Fibrochondrocyte	Primary fibrochondrocytes	tailored biomimetic surface (C6S surface) on glass cover slip	tailored biomimetic surface on PLGA scaffolds	Human fibrochondrocyte redifferentiation was enhanced by hypoxia in the 3D cultures, independent of hypoxia inducible factor (HIF) transcriptional activity and was shown to potentially involve the transcriptional activation of Sox-9.	[93]
Hepatocyte	Primary rat hepatocytes	Collagen-coated polymeric substrates	3D polymeric scaffold coated with collagen	Albumin and urea assays demonstrated that hepatocytes cultured in the 3D scaffold maintained higher levels of liver specific function over a period of 6 days as compared to the monolayer control. These results may be attributed to the high local concentration of soluble factors within the scaffold, which is important for maintaining the hepatocyte phenotype.	[94]
Hepatocyte	Hepatocyte cell line HepG2	Monolayer	Agar/gelatin sponge	The results showed that the agar–gelatin hybrid sponges induced the formation of 3D HepG2 spheroids with significant liver-specific functions. These spheroids exhibited higher amounts of albumin and urea synthesis than the control monolayer culture. These 3D spheroids were found to be more sensitive to the drug than the control monolayer.	[95]
Myoblast	Rat skeletal myoblasts	Culture plate	PLGA-collagen composite scaffolds	Tensile strain induces higher and faster integrin β1 and ILK expression in 3D cultured rat skeletal myoblasts than in 2D cultures.	[96]
Osteoblast	Rat bone marrow cells	Monolayer	3D homogenized pellet and 3D organotypic explant	The 3D organotypic marrow explant culture resulted in the greatest level of ossification with plate-like bone formations.	[97]
Osteoblast	Osteoblast-like cells MG-63	Monolayer on culture plates	Solid PLGA microspheres	The microspheres give stronger cell attachment, better phenotypic characteristics, higher cell viability and mineralization levels but lower cell count compared with 2D monolayer cell culture over 28 day cell culture studies.	[98]
Tenocyte	Human primary tenocytes	Monolayer	PLGA scaffolds or high-density cultures	Compared to native tendon, decorin and COMP were reduced in 2D and increased in 3D culture almost to *ex vivo* level.	[99]
Tenocyte	Rat dermal fibroblasts	Monolayer	3D resorbable polyester scaffolds	Direct comparisons between the 2D and 3D studies are difficult, given the differences in experimental variables (*i.e.* surface chemistry, topography, serum content, seeding density, *etc.*).	[100]

a PRP: platelet-rich plasma, ECM: extracellular matrix, PLGA: poly (lactic-co-glycolic) acid, COMP: a typical non-collagenous protein component of tendon ECM.

### A1. Surface Porosity Estimation from SEM Photos

In our report, we estimate the surface porosity of the materials from SEM photos using procedures modified from a method developed by Abdullah *et al.* [101].

#### A1.1. Principle of Estimation

This method estimates the surface porosity by calculating the volume fraction of solid under the surface observed in SEM photos to the total volume of space involved in the surface. The z-value was obtained through converting the intensity of each pixel in the photos into a numerical value through function in the software OriginPro.

#### A1.2. Photo Selection

In order to obtain more reliable estimates, we select SEM photos for the surface porosity estimation based on the following criteria.

- Low magnification (30× photos for our case, to ensure there is a sufficient number of pores/craters in the photos).
- No charging of the materials in the photos (charge build up during SEM examination would cause bright spots on the photos which may result in extremely high intensity values that does not reflect the true topography).
- Good contrast and brightness (the photos should have appropriate contrast so that the intensity of each pixel of the photos truly represents the surface topography).

#### A1.3. Modifications

- The lowest z-value mapped from our photos is not always 0. Thus we made a slight modification in the method to accurately estimate the porosity for our porous materials. In addition to obtaining the f_max_ value, we also incorporated f_min_ into the calculation. Considering a surface represented by a function f(x,y),
  - Surface porosity $\left(\phi \right)=1-\frac{V_{\text{solid}}}{V_{\text{total}}}$
  - $V_{\text{solid}}=\int_{y_{\min}}^{y_{\max}}\int_{x_{\min}}^{x_{\max}}f\left(x,y\right)\text{dxdy}-f_{\min}\left(x_{\max}-x_{\min}\right)\left(y_{\max}-y_{\min}\right)$
  - $V_{\text{total}}=\left(f_{\max}-f_{\min}\right)\left(x_{\max}-x_{\min}\right)\left(y_{\max}-y_{\min}\right)$
- The labels on the photos were cropped away. Otherwise, they would be treated as topography features.
- When the photos in TIFF format were mapped into the pixel intensity values using OriginPro 8.5.1 (Image > Conversion > Convert to Data), the first and last x/y values may be extremely small. In such case, the value needs to be reset according to the matrix dimension (Matrix > Set Dimension and Labels). Otherwise, the Integral values obtained may become 0 since extremely small.
- Contour maps were produced to check whether the numerical values representing the surface topography is consistent with the SEM photos (Plot > Contour > Color Fill).

#### A1.4. Note

The surface porosity estimated using this method may be slightly lower than the bulk porosity values obtained using other methods as this method assumes the volume under the surface observed in SEM is solid completely which in fact pores may be present in the volume. On the other hand, as the porosity values estimated from image analysis may be affected by the contrast of photos, we estimate the surface porosities as supplementary information to the bulk porosity estimated by the Archimedes drainage method.

### A2. ALP Activity Due to Adsorbed ALP from Serum

We did not draw conclusion from ALP activity assay in this report since there was high ALP activity for calcium phosphate samples even for “no cell” control (all conditions were the same except cells were not added and thus supposed not to give ALP activity) (Figure 6). Thus we investigated whether cell culture medium may contribute to the detected ALP activity. From Figure A1, the ALP activity increases linearly with the volume of medium used in the assay, indicating that the medium contains component(s) responsible for the ALP activity. Comparing the activity of medium of different serum concentration, it can be shown that the serum has ALP activity.

In order to remove non-specific proteins, a washing step is usually performed to remove medium before the addition of substrate solution for ALP. However, the adsorption of protein on materials may actually not be weak and not easily removed by washing alone. For instance, in ELISA (Enzyme-linked immunosorbent assay), the antigen can be coated on the assay plate by adsorption alone and remain on the ELISA plate after washing [35,36]. Different washing methods were tested to get rid of the adsorbed ALP but the results were not satisfactory (data not shown).

For ALP enzymatic assay, some studies change to lower serum concentration for the cell culturing before the assay [37,38,39], use charcoal-stripped FBS [40] or charcoal-dextran-treated FBS [41]. ITS supplement (which includes insulin, transferrin and selenium) has also been used to substitute serum for a study on calcium phosphate particles [42]. Insulin stimulates cell growth and other classical insulin responses such as increased fatty acid and glycogen synthesis are seen in serum-free medium. Transferrin was identified as an essential growth factor for many cell types. Selenium is very often necessary for optimal cell growth (Roche ITS supplement information). However, the modifications may not totally avoid ALP adsorption problem. For example, even when the serum-containing cell culture medium was changed to serum-free or low serum medium, the adsorbed ALP on the scaffold surface may not be desorbed effectively. The results may still be interfered by the adsorbed ALP on the materials as the surface area among different porous scaffolds may have much larger variation compared with tissue culture plate, causing variations in results.

It may be justified if the ALP activity of osteoblasts is much higher than that present in the serum. Thus the activity of serum ALP was compared with the ALP activity of differentiated 10T1/2 cells. By comparing the ALP activity of serum to that of 10T1/2 cells which has been induced to be differentiated into osteoblast by recombinant human bone morphogenetic protein 2 (BMP2), dexamethasone and *L*-ascorbic acid treatment for 3 days, the ALP activity in 0.1% FBS is approximately equal to the ALP activity of 10,000 induced 10T1/2 cells (data not shown). This number is significant compared to the number of cells plated, indicating that the ALP activity due to the serum cannot be ignored. Similarly, care should be taken when osteocalcin (OCN) is monitored using similar procedures as OCN is also present in serum. For calcium phosphate materials, “no cell” control is required to assess the background ALP activity resulted from serum.

### A3. Base of Normalization for ALP Enzymatic Assay

On the other side, different numbers of cells can be present on materials of different porosities in the same volume of disc. Thus we hypothesized that the difference in the base of normalization may be one of the reasons of the opposite conclusions. As illustrated in Figure A2(a), an increase in the total ALP activity can be resulted from cell proliferation, higher degree of osteo-differentiation and cell growth. And more bone would be formed as a result of these events. Thus ALP activity can reflect bone formation. Nevertheless, for assessing osteoinductivity which is the ability of the material to induce undifferentiated cells into the osteo-lineage, normalized (specific) ALP activity should be used. Several common bases of normalization for ALP activity are protein amount [43,44,45,46,47,48,49], DNA amount [50,51,52,53], cell number [54,55,56], material surface area [57], construct [34,58], lysate volume [59,60] and tissue amount [61]. Some parameters may change greatly (to nearly two fold) during the cell cycle of proliferating cells as shown in Figure A2(b). In the G1 and G2 phases, the cells would synthesize proteins and increase in size while in the S phase, the cells would synthesize DNA, causing it to double in amount. After the M phase, the cells would be separated into two daughter cells with all components evenly distributed between the two and the cell number would increase. When the cells have less space for growth at high cell density, they may divide with less time spending in the G1 and G2 phases, resulting in smaller cells. Yet the S and M phases would still be present. Thus the amount of DNA per cell would still be the same. As a consequence, the cell density difference would cause difference in normalization with reference to different cell attributes such as DNA amount, protein amount, cell number, *etc.*

In the study of different cell plating densities, we measured the protein amount for normalization as ALP is an enzyme (protein that has catalyst function) that is present on the cell membrane and cytosol. Our result shows that the protein amount of cells at day 7 is roughly the same for the three chosen cell plating densities (1 × 10^4^, 2 × 10^4^ and 4 × 10^4^ cells per well of a 96-well plate). Besides, the areas covered by cells are the same for different initial cell plating densities (Figure 4c). Therefore, the higher cell plating density would result in higher specific ALP activity no matter normalized with reference to protein amount or area of coverage. Figure A2(c) compares the bases of normalization at low and high cell density. At higher cell density, the amount of DNA would be greater even though the cell “area” or “volume” and protein amount may be similar. Hence, when we normalized the ALP activity with reference to the cell number or DNA amount, the specific ALP activity of high cell plating density might be lower than low cell plating density as the bases of normalization increases with high cell plating density. As a result, the conclusions can be different for different bases of normalization for the ALP activity and we should analyze the data while keeping the possible effects in mind or further validate it using RT-qPCR experiments.
